# DeepLN: A Multi-Task AI Tool to Predict the Imaging Characteristics, Malignancy and Pathological Subtypes in CT-Detected Pulmonary Nodules

**DOI:** 10.3389/fonc.2022.683792

**Published:** 2022-05-11

**Authors:** Chengdi Wang, Jun Shao, Xiuyuan Xu, Le Yi, Gang Wang, Congchen Bai, Jixiang Guo, Yanqi He, Lei Zhang, Zhang Yi, Weimin Li

**Affiliations:** ^1^ Department of Respiratory and Critical Care Medicine, Med-X Center for Manufacturing, West China Hospital, West China School of Medicine, Sichuan University, Chengdu, China; ^2^ Machine Intelligence Laboratory, College of Computer Science, Sichuan University, Chengdu, China; ^3^ Precision Medicine Center, West China Hospital, Sichuan University, Chengdu, China; ^4^ Department of Medical Informatics, West China Hospital, Sichuan University, Chengdu, China

**Keywords:** pulmonary nodules, pathological subtypes, artificial intelligence, deep learning, computed tomography

## Abstract

**Objectives:**

Distinction of malignant pulmonary nodules from the benign ones based on computed tomography (CT) images can be time-consuming but significant in routine clinical management. The advent of artificial intelligence (AI) has provided an opportunity to improve the accuracy of cancer risk prediction.

**Methods:**

A total of 8950 detected pulmonary nodules with complete pathological results were retrospectively enrolled. The different radiological manifestations were identified mainly as various nodules densities and morphological features. Then, these nodules were classified into benign and malignant groups, both of which were subdivided into finer specific pathological types. Here, we proposed a deep convolutional neural network for the assessment of lung nodules named DeepLN to identify the radiological features and predict the pathologic subtypes of pulmonary nodules.

**Results:**

In terms of density, the area under the receiver operating characteristic curves (AUCs) of DeepLN were 0.9707 (95% confidence interval, CI: 0.9645-0.9765), 0.7789 (95%CI: 0.7569-0.7995), and 0.8950 (95%CI: 0.8822-0.9088) for the pure-ground glass opacity (pGGO), mixed-ground glass opacity (mGGO) and solid nodules. As for the morphological features, the AUCs were 0.8347 (95%CI: 0.8193-0.8499) and 0.9074 (95%CI: 0.8834-0.9314) for spiculation and lung cavity respectively. For the identification of malignant nodules, our DeepLN algorithm achieved an AUC of 0.8503 (95%CI: 0.8319-0.8681) in the test set. Pertaining to predicting the pathological subtypes in the test set, the multi-task AUCs were 0.8841 (95%CI: 0.8567-0.9083) for benign tumors, 0.8265 (95%CI: 0.8004-0.8499) for inflammation, and 0.8022 (95%CI: 0.7616-0.8445) for other benign ones, while AUCs were 0.8675 (95%CI: 0.8525-0.8813) for lung adenocarcinoma (LUAD), 0.8792 (95%CI: 0.8640-0.8950) for squamous cell carcinoma (LUSC), 0.7404 (95%CI: 0.7031-0.7782) for other malignant ones respectively in the malignant group.

**Conclusions:**

The DeepLN based on deep learning algorithm represented a competitive performance to predict the imaging characteristics, malignancy and pathologic subtypes on the basis of non-invasive CT images, and thus had great possibility to be utilized in the routine clinical workflow.

## Introduction

Lung cancer is the most commonly diagnosed cancer (11.6% of all cases) and the leading cause of cancer-related deaths (18.4% of the total cancer deaths) globally (1). The wide application of computed tomography (CT) in routine clinical practice has enabled lung cancer detection and intervention at a relatively early stage, markedly improving the survival outcomes of the whole patient population. There have been widely known lung cancer screening programs such as the National Lung Screening Trial (NLST) and Nederlands–Leuvens Longkanker Screenings Onderzoek (NELSON) demonstrating a reduction in the lung-cancer mortality with low-dose computed tomography (LDCT) screening of about 20% as compared with the mortality in the chest radiography group (2–4). However, more than 96% of all positive screens detected by LDCT were false positives (2). Therefore, despite the observed contribution of the LDCT screening in improving the prognosis of certain patients with early confirmed lung cancer, this method can also cause deleterious effects in the patient cohorts with benign lung nodules or even without any diseases, such as unnecessary investigations, increased anxiety and high financial expenses. At a time when healthcare resources are generally limited, considering the heterogeneous manifestations of medical imaging methods, a more rapid thorough interpretation of the imaging results will entail to better grasp the biological nature of lung nodules.

An in-depth interpretation requires a wide coverage of multidimensional characteristics. There have been several classical guidelines (e.g., Lung‐RADS, Fleischner Society 2017, clinical practice consensus guidelines for Asia) that consider elements including but not limited to nodule size and the density presented on CT images in classification (5–7). And the morphology of nodules, with features such as spiculation, lobulation, pleural indentation and lung cavity, has also been reported to have a clear positive relationship with the risk of malignancy (8). However, the manual classification task by human visual analyses is tedious and time-consuming, and it highly depends on the clinical experience of the doctors, resulting in substantial inter-observer variability, even among experienced radiologists (9). In areas where the adequate experienced thoracic radiologists are unavailable, this problem becomes considerably more severe. Therefore, computer-based techniques that could provide potential assistance to clinical decision making in evaluating the imaging results, defining the sub-classifications, and eventually predicting the malignancy risks of lung nodules would be of significant value.

Deep learning, a subset of machine learning, has achieved impressive results with accuracy at least equivalent to expert physicians in several medical image classification tasks such as grading of diabetic retinopathy, assessment of skin lesions as benign or malignant, and detection of lymph node metastasis in breast cancer (10–13). In the field of respiratory diseases, deep learning algorithms have also been utilized and trained to detect the pulmonary nodules, to predict the cancer risk of nodules, and to assess the tumor invasiveness especially in adenocarcinoma, the most common subtype of lung cancer (14–16). However, investigations digging into the classification of more specific subtypes within benign and malignant pulmonary nodules with deep learning methods were relatively limited. However, due to the heterogeneous pathologic nature and continuously evolving characteristics of these nodules, finer taxonomy should be fed into the deep learning algorithms to make this advanced technical modality better fit with routine clinical practice, which thus became the goal we sought to achieve in the present study.

Previously, we proposed a novel deep learning system for the assessment of lung nodules named DeepLN for screening throax pathologies to identify the location and general nature of lung nodules, which presented a favorable performance (17, 18). In the current study, we extended this deep learning algorithm (DeepLN) to account for a wide range of radiological manifestations like the tumor densities and the morphologic features, predict the pathological subtypes including the benign status (benign tumors, inflammation, other lesions) and the malignant status (adenocarcinoma, squamous cell carcinoma, other types). Our developing, training, and test procedures were all based on a large real-world CT-related images dataset.

## Materials and Methods

### Patients Population and Nodules Dataset

We built a retrospective cohort comprising 8950 nodules from 5823 patients at the West China Hospital of Sichuan University. Patients of older than 18 years old, with radiological examination reporting pulmonary nodules were included. Nodules were only included if they had a diagnosis confirmed by pathology. Pathological results were regarded as the gold standard for clinical diagnoses, which could distinguish malignant nodules from the benign ones. Furthermore, the pathological results were used to distinguish various subtypes of benign and malignant nodules. Benign nodules were divided into benign tumors, inflammatory nodules, and other benign lesions, whereas malignant nodules were classified as lung adenocarcinoma (LUAD), squamous cell carcinoma (LUSC), and other cancer types. In our study, all nodule tissues were collected after surgery or biopsy. Ethics approval was obtained from the ethics committee of West China Hospital, Sichuan University.

The latest preoperative chest CT images of the whole cohort were collected after anonymization in Digital Imaging and Communications in Medicine (DICOM) format, including both thin-section (1-3 mm) and thick-section (5mm) scans. Scanning parameters were set according to the operating specifications: 120 kV tube voltage, 200-500 mA tube current, 0.4-0.7 s rotation time, 512 × 512 pixel matrix.

First, the nodules on CT images were annotated by junior radiologists and were reviewed by senior radiologists, with the assistance of a semi-automatic annotation system constructed previously (19). The characteristics annotated by radiologists in our dataset included density (pure-ground glass opacity called pGGO, mixed-ground glass opacity named mGGO, solid) and morphology (spiculation, lobulation, pleural indentation, and lung cavity). Biases were minimized with the final annotations coming from the consensus from 2 senior radiologists. The whole dataset was randomly divided into a training set (70%), a validation set (10%), and a test set (20%).

### Multi-Task DeepLN Architecture

**Figure 1:**
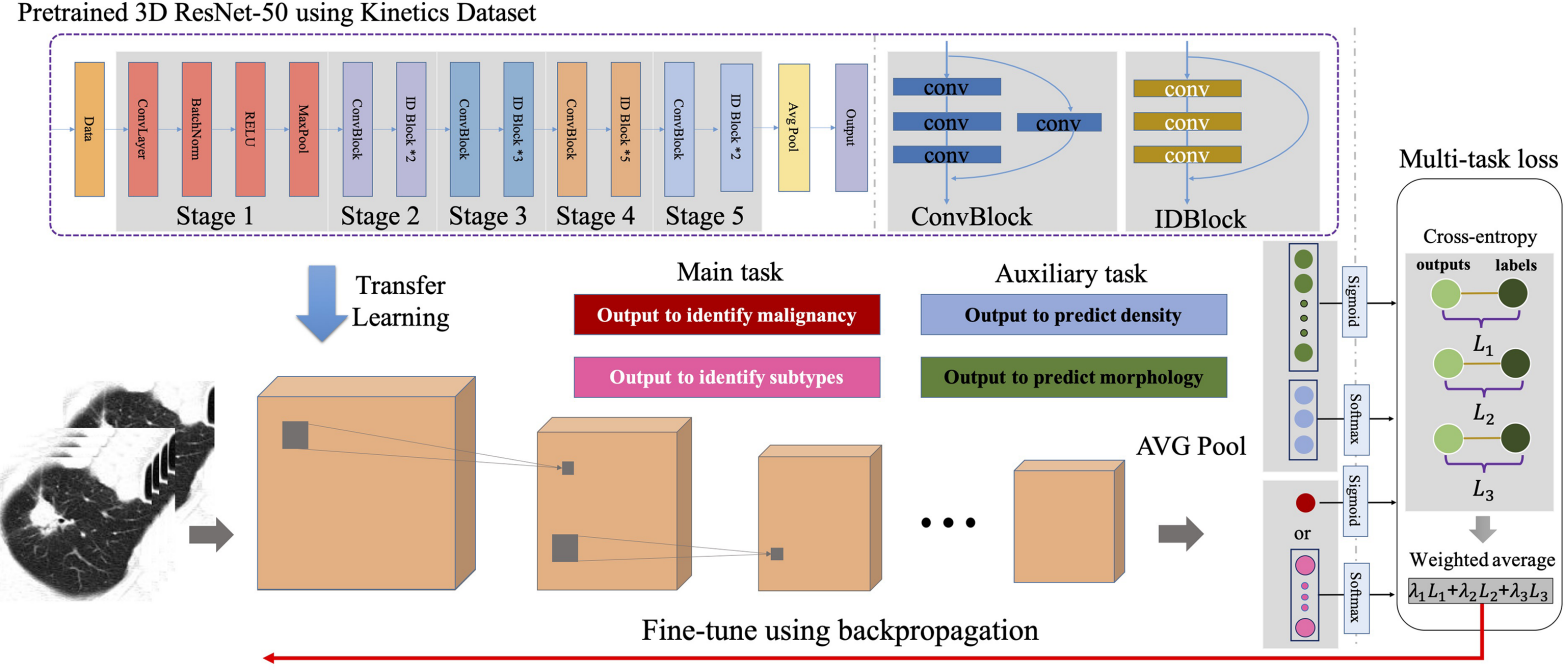
Architecture of our deep learning model.

We built deep learning models for classifying benign/malignant nature, and pathological subtypes assisted by the auxiliary tasks of nodule imaging feature classification. The architecture of our model was shown in **Figure 1**, where we used 3D-ResNet as the backbone network. To be started, all the nodules were cropped into 20×96×96 patches from the raw images according to the nodules’ center, and then the patches were enlarged to 32×128×128. Besides, the CT values were clipped in the range of [-1300, 500] and normalized to [0, 1]. Before feeding to the networks, the patches were converted to tensors with the channel of 1, and several data augmentation operations, involving random flip, rotation and center location perturbation, were conducted. The 3D-ResNet backbone computed and further extracted the discriminative features, and in the output phase, the last fully-connected (FC) layer was placed to obtain the final classification probabilities using sigmoid for binary classification tasks and softmax for multi-class classification tasks. The 3D-ResNet backbone (20), which inherited the nature of ResNet (21) and was modified for three-dimensional input, was stacked by a 3D convolutional (Conv) layer, a max-pooling (MP) layer, four 3D residual blocks (ResBlocks), and an average-pooling (AP) layer (20). The residual block, as the key component of ResNet, was a strong feature extractor and utilized the shortcut connections to effectively train the deep neural networks while maintaining fast convergence. Each block consisted of several convolutional layers with different parameters. The residual block could be categorized into two types according to the types of shortcut connection. The shortcut connection in IDBlock was an identify mapping, while it was an 1×1×1 convolution mapping in ConvBlocks. The max-pooling layer served as a downsampling function and was used to generate high-level features. Since it was reported that deep learning models were capable of transferring image representations from large-scale datasets, the constructed model was facilitated by the pretrained parameters based upon Kinetics (21, 22), an action recognition dataset that commonly used for 3D convolutional neural network pretraining. More specifically, we initialized the parameters of four ResBlocks using network parameters that had been converged at the Kinetics dataset (20).

Referred to Lung‐RADS, in which nodules size, density and morphology could be employed to identify the malignancy of lung nodules, we employed lung nodule density and morphology as auxiliary labels to train the proposed model sufficiently using multi-task learning. Moreover, these morphology features also served as auxiliary tasks for classifying pulmonary nodule pathological subtypes. As shown in **Figure 1**, for the main task, we employed one neuron to output the probability of the nodule being malignancy or six neurons to output the probabilities of the nodule belonging to a certain pathological subtype. For the auxiliary tasks, three neurons were employed to output the probabilities of the nodule density, namely pGGO, mGGO and solid, and four neurons were employed to output the probabilities of four characteristics of morphology. These auxiliary tasks would provide more feedback and reduce the certain noisy patterns regarding the single task, so that more general representations of pulmonary nodules could be extracted from the networks and facilitate more accurate prediction. The DeepLN learning framework was guided by the multi-task loss function, which was the weighted combination of the binary cross-entropy (BCE) loss and softmax cross-entropy (SCE) loss. BCE loss was used for binary classification tasks, namely the benign/malignant classification and characteristic classification. SCE loss was used for multi-class classification tasks, namely the pathological subtypes classification and density classification. Supposing $L_{BCE}^{bm},\;L_{SCE}^{subtype},\;L_{SCE}^{den},\;L_{BCE}^{morph}$ denoted the loss functions of the benign/malignancy classification, pathological subtype classification, nodule density classification, and morphology characteristic classification task, respectively. The multi-task learning loss functions were defined as follows:


$$L_{1}=\lambda_{1}L_{BCE}^{bm}+\;\lambda_{2}L_{SCE}^{den}+\;\lambda_{3}L_{BCE}^{morph}$$

$$L_{2}=\lambda_{1}L_{SCE}^{subtype}+\lambda_{2}L_{SCE}^{den}+\lambda_{3}L_{BCE}^{morph}$$

Here, λ denoted the weight factors of the classification tasks. As can be seen, benign/malignancy and pathological subtype classification were respectively regarded as the main task, and the nodule density and characteristic classification were regarded as the assisted tasks. We chosed *λ*_1_ = 0.4, *λ*_2_ = 0.2, and *λ*_3_ = 0.4 in *L*_1_, and since the subtype classification was a more difficult task than benign/malignancy classification, we selected a larger value of *λ*_1_, i.e., *λ*_1_ = 0.8, *λ*_2_ = 0.05, and *λ*_3_ = 0.15 in *L*_2_.

### Implementation and Training Details

The training experiments were conducted with Pytorch (v.1.0.0) on the Red Hat 4.8.5 server with one NVIDIA Tesla V100 GPU (32GB). To reach the best training result, the hyper-parameters of the models were carefully tuned by 100 epochs, with a learning rate of 0.001, a weight decay of 0.001, and a momentum of 0.9. The learning rate would be multiplied by 0.1 if the error in validation set did not reduce in 20 epochs. To accelerate and optimize the training progress, mini-batch stochastic gradient descent was adopted, with a batch size of 32.

### Evaluation Metrics

The performance of each trained model was evaluated in validation and test sets from dimensions of its accuracy (ACC), recall, precision, specificity, F1 score, the area under the curve (AUC) and 95% confidence interval (CI). To ensure best practice in application of AI in medical imaging, CLAIM (Checklist for AI in Medical Imaging) was rigorously applied (**Supplementary Table 1**) (23).

## Results

### Demographic and Clinical Characteristics

**Figure 2:**
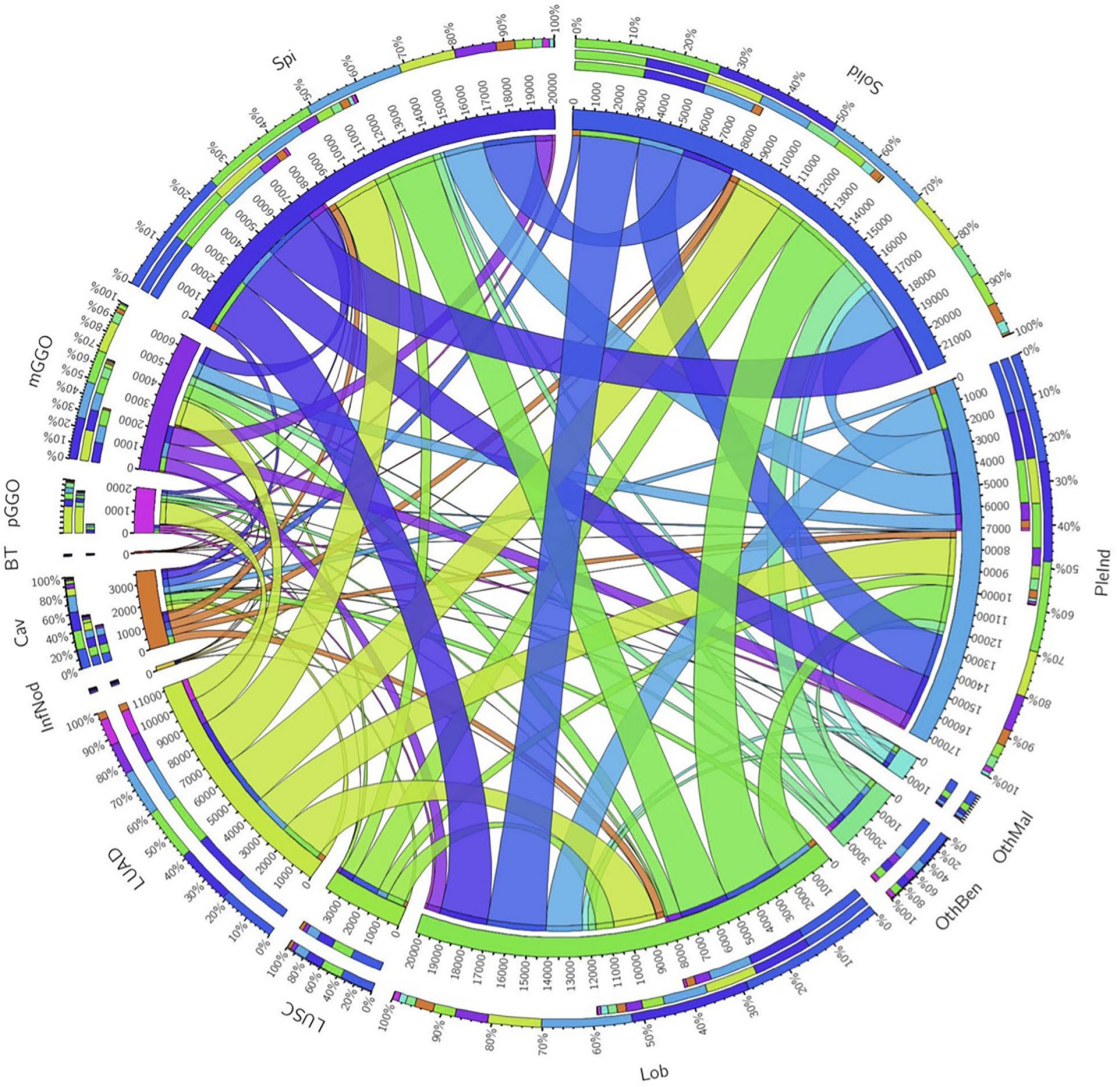
Circos of the correlation of pathological subtypes and morphological features. Outermost circle denoted the total number of corresponding relationships, the middle and innermost circle denoted the relationship. The wider the strip, the stronger the correlation. Spi, Spiculation; Lob, Lobulation; PleInd, Pleural Indentation; Cav, Cavity; pGGO, pure ground glass opacity; mGGO, mixed ground glass opacity; BT, Benign Tumors; InfNod, Inflammatory Nodule; OthBen, Other Benign Lesions; LUAD, Adenocarcinoma; LUSC, Squamous Carcinoma; OthMal, Other Malignant Tumors.

In total, 2211 benign nodules and 6739 malignancy nodules from 5823 patients were included (**Table 1**). Adenocarcinoma was the most prevalent subtype with 4666 cases. The majority density of nodules was solid, accounting for 62% of malignant nodules and 68% of benign lesions. Spiculation (46%) and lobulation (49%) were common features of malignant nodules. Circos of nodules characteristics illustrated the correlation of pathological subtypes and morphological features (**Figure 2**). For example, adenocarcinoma occupied the most samples of all the cases and closely related to lobulation, spiculation, lung cavity and solid. Lobulation always appeared with solid and spiculation at the same time, which indicated that imaging manifestation features might assist the diagnosis of subtypes.

**Table 1:** Characteristics of included patients and nodules dataset.

	Malignancy No. (%)	Benignancy No. (%)
**Patients, No**	4384	1439
**Age, mean (SD)**	58.59 (10.27)	57.90 (10.29)
**Sex**		
Male	2522 (42)	798 (55)
Female	1862 (58)	641 (45)
**Smoking status**		
Former or Current	2036 (46)	80 (6)
Never	2216 (51)	131 (9)
Unknown	132 (3)	1228 (85)
**Nodules, No**	6739	2211
**Subtypes**		
Benign Tumor	–	695(31)
Inflammatory Nodule	–	1106(50)
Other Benign Lesions	–	410(19)
Adenocarcinoma	4666 (69)	–
Squamous Carcinoma	1366 (20)	–
Other Malignant Tumors	707 (11)	–
**Density**		
pGGO	1089 (16)	251 (11)
mGGO	1474 (22)	457 (21)
Solid	4176 (62)	1503 (68)
**Nodule Feature**		
Spiculation	3111 (46)	426 (19)
Lobulation	3297 (49)	400 (18)
Pleural Indentation	2520 (37)	498 (23)
Lung Cavity	470 (7)	32 (14)

SD, Standard Deviation; pGGO, pure-ground glass opacity; mGGO, mixed-ground glass opacity.

### Feature Detection Performance

**Figure 3:**
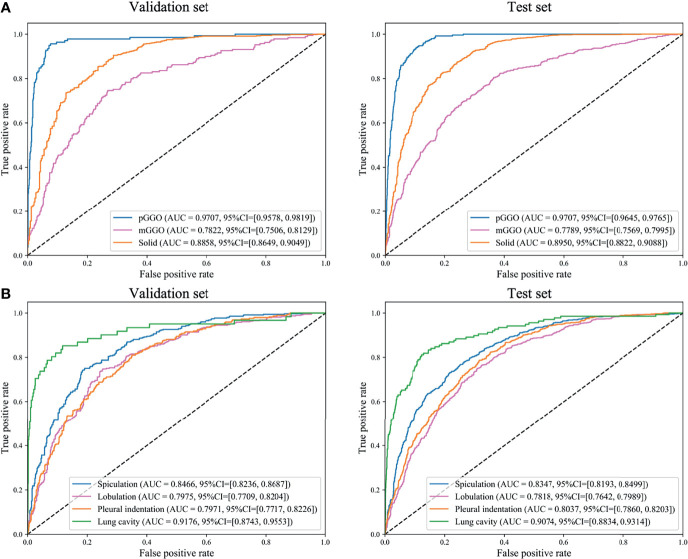
Receiver operating characteristics (ROC) curves of DeepLN to identify, **(A)** density, **(B)** morphology of nodules. AUC, area under curve; pGGO, pure-ground glass opacity; mGGO, mixed-ground glass opacity.

The density of nodules was of paramount importance for clinicians. The AUCs of pGGO nodules were 0.9707 (95% CI: 0.9578-0.9819), 0.9707 (95% CI: 0.9645-0.9765) in the validation set and the test set respectively (**Table 2** and **Figure 3**). The AUCs of solid nodules were 0.8858 (95%CI: 0.8649-0.9049) and 0.8950 (95%CI: 0.8822-0.9088) in the validation and test set. Nevertheless, the identification performance of the model for mGGO was relatively poor with an AUC of 0.7822 (95%CI: 0.7506-0.8129) in validation set and 0.7789 (95%CI: 0.7569-0.7995) in test set. The confusion matrix was shown in **Supplementary Figure 1**.

**Table 2:** Performance of DeepLN to predict the imaging characteristics, malignancy, and pathological subtypes.

Characteristics	Validation Set						Test Set					
	ACC(95%CI)	Sensitivity(95%CI)	Precision(95%CI)	Specificity(95%CI)	F1 score(95%CI)	AUC(95%CI)	ACC(95%CI)	Sensitivity(95%CI)	Precision(95%CI)	Specificity(95%CI)	F1 score(95%CI)	AUC(95%CI)
**Imaging Characteristics**												
**Density**												
pGGO	0.7897 (0.7685, 0.8110)	0.8406 (0.7829, 0.8897)	0.8169 (0.7639, 0.8675)	–	0.8286 (0.7863, 0.8660)	0.9707 (0.9578, 0.9819)	0.7902 (0.7743, 0.8067)	0.8080 (0.7663, 0.8492)	0.7652 (0.7195, 0.8062)	–	0.7860 (0.7506, 0.8185)	0.9707 (0.9645, 0.9765)
mGGO		0.3810 (0.3263, 0.4332)	0.5581 (0.4839, 0.6242)	–	0.4528 (0.3960, 0.5031)	0.7822 (0.7506, 0.8129)		0.3711 (0.3300, 0.4122)	0.5618 (0.5103, 0.6125)	–	0.4469 (0.4066, 0.4871)	0.7789 (0.7569, 0.7995)
Solid		0.9136 (0.8936, 0.9328)	0.8315 (0.8065, 0.8553)	–	0.8706 (0.8532, 0.8876)	0.8858 (0.8649, 0.9049)		0.9274 (0.9155, 0.9402)	0.8416 (0.8243, 0.8587)	–	0.8824 (0.8713, 0.8941)	0.8950 (0.8822, 0.9088)
**Nodule Feature**												
Spiculation	0.7823 (0.7596, 0.8061)	0.7224 (0.6815, 0.7606)	0.7307 (0.6907, 0.7686)	0.8223 (0.7943, 0.8487)	0.7265 (0.6940, 0.7558)	0.8466 (0.8236, 0.8687)	0.7594 (0.7443, 0.7772)	0.7166 (0.6894, 0.7448)	0.7023 (0.6766, 0.7305)	0.7890 (0.7700, 0.8097)	0.7094 (0.6885, 0.7306)	0.8347 (0.8193, 0.8499)
Lobulation	0.7438 (0.7200, 0.7676)	0.7247 (0.6854, 0.7618)	0.6992 (0.6626, 0.7366)	0.7586 (0.7300, 0.7910)	0.7117 (0.6816, 0.7400)	0.7975 (0.7709, 0.8204)	0.7130 (0.6957, 0.7314)	0.6653 (0.6373, 0.6920)	0.6513 (0.6235, 0.6805)	0.7469 (0.7243, 0.7696)	0.6582 (0.6352, 0.6813)	0.7818 (0.7642, 0.7989)
Pleural Indentation	0.7426 (0.7188, 0.7676)	0.5898 (0.5404, 0.6385)	0.6214 (0.5714, 0.6693)	0.8194 (0.7926, 0.8473)	0.6052 (0.5625, 0.6427)	0.7971 (0.7717, 0.8226)	0.7337 (0.7180, 0.7504)	0.6019 (0.5691, 0.6320)	0.6404 (0.6068, 0.6739)	0.8084 (0.7899, 0.8276)	0.6205 (0.5937, 0.6463)	0.8037 (0.7860, 0.8203)
Lung Cavity	0.9603 (0.9501, 0.9705)	0.5902 (0.4918, 0.6949)	0.7826 (0.6818, 0.8810)	0.9878 (0.9807, 0.9939)	0.6729 (0.5833, 0.7568)	0.9176 (0.8743, 0.9553)	0.9470 (0.9380, 0.9559)	0.4420 (0.3750, 0.5113)	0.7722 (0.6944, 0.8462)	0.9891 (0.9847, 0.9932)	0.5622 (0.4974, 0.6293)	0.9074 (0.8834, 0.9314)
**Malignancy**												
Single-Task	0.8333 (0.8129, 0.8549)	0.9145 (0.8959, 0.9319)	0.8714 (0.8509, 0.8926)	0.5814 (0.5300, 0.6414)	0.8925 (0.8773, 0.9078)	0.8636 (0.8412, 0.8866)	0.8269 (0.8118, 0.8414)	0.9142 (0.9023, 0.9266)	0.8643 (0.8490, 0.8782)	0.5581 (0.5189, 0.5959)	0.8886 (0.8783, 0.8987)	0.8361 (0.8172, 0.8542)
Multi-Task	0.8356 (0.8152, 0.8560)	0.9250 (0.9081, 0.9413)	0.8666 (0.8450, 0.8876)	0.5581 (0.5025, 0.6143)	0.8949 (0.8802, 0.9086)	0.8696 (0.8468, 0.8911)	0.8331 (0.8185, 0.8465)	0.9275 (0.9152, 0.9388)	0.8619 (0.8474, 0.8756)	0.5421 (0.5033, 0.5779)	0.8935 (0.8834, 0.9034)	0.8503 (0.8319, 0.8681)
**Subtype classification (Multi-task)**												
Benign Tumors	0.6435 (0.6159, 0.6700)	0.3385 (0.2459, 0.4500)	0.5238 (0.3953, 0.6538)	–	0.4112 (0.3089, 0.5149)	0.8436 (0.8013, 0.8885)	0.6499 (0.6311, 0.6699)	0.4825 (0.4067, 0.5520)	0.6053 (0.5321, 0.6860)	–	0.5370 (0.4715, 0.5978)	0.8841 (0.8567, 0.9083)
Inflammatory Nodules		0.3675 (0.2889, 0.4444)	0.4674 (0.3750, 0.5542)	–	0.4115 (0.3299, 0.4810)	0.8331 (0.8011, 0.8627)		0.3349 (0.2844, 0.3889)	0.4828 (0.4138, 0.5484)	–	0.3955 (0.3402, 0.4471)	0.8265 (0.8004, 0.8499)
Other Benign Lesions		0.1000 (0.0286, 0.1818)	0.1333 (0.0385, 0.2414)	–	0.1143 (0.0370, 0.2105)	0.8073 (0.7556, 0.8533)		0.0921 (0.0441, 0.1528)	0.2593 (0.1304, 0.4167)	–	0.1359 (0.0667, 0.2157)	0.8022 (0.7616, 0.8445)
Adenocarcinoma		0.8971 (0.8733, 0.9196)	0.7291 (0.6982, 0.7584)	–	0.8044 (0.7819, 0.8247)	0.8618 (0.8414, 0.8810)		0.9044 (0.8872, 0.9205)	0.7226 (0.7027, 0.7436)	–	0.8033 (0.7867, 0.8189)	0.8675 (0.8525, 0.8813)
Squamous Carcinoma		0.5231 (0.4545, 0.5985)	0.5913 (0.5169, 0.6667)	–	0.5551 (0.4936, 0.6166)	0.8994 (0.8786, 0.9200)		0.5423 (0.4947, 0.5897)	0.5600 (0.5095, 0.6111)	–	0.5510 (0.5075, 0.5915)	0.8792 (0.8640, 0.8950)
Other Malignant Tumors		0.1471 (0.0800, 0.2222)	0.3448 (0.2000, 0.5000)	–	0.2062 (0.1163, 0.2979)	0.7212 (0.6687, 0.7709)		0.1288 (0.0827, 0.1778)	0.3148 (0.2143, 0.4200)	–	0.1828 (0.1222, 0.2449)	0.7404 (0.7031, 0.7782)
**Subtype classification (Single-task)**												
Benign Tumors	0.6501 (0.6236, 0.6744)	0.3692 (0.2698, 0.4714)	0.5000 (0.3750, 0.6250)	–	0.4248 (0.3191, 0.5192)	0.8386 (0.7952, 0.8795)	0.6300 (0.6100, 0.6477)	0.4476 (0.3776, 0.5133)	0.5333 (0.4615, 0.6017)	–	0.4867 (0.4228, 0.5434)	0.8573 (0.8262, 0.8852)
Inflammatory Nodule		0.3932 (0.3232, 0.4632)	0.5287 (0.4375, 0.6170)	–	0.4510 (0.3787, 0.5171)	0.8348 (0.8023, 0.8623)		0.3493 (0.2965, 0.4000)	0.4294 (0.3632, 0.4880)	–	0.3852 (0.3298, 0.4327)	0.8048 (0.7747, 0.8318)
Other Benign Lesions		0.0750 (0.0000, 0.1515)	0.1667 (0.0000, 0.3158)	–	0.1034 (0.0357, NA)	0.8032 (0.7404, 0.8602)		0.0921 (0.0423, 0.1538)	0.2333 (0.1111, 0.3684)	–	0.1321 (0.0612, 0.2056)	0.7853 (0.7474, 0.8280)
Adenocarcinoma		0.8951 (0.8708, 0.9177)	0.7166 (0.6878, 0.7462)	–	0.7960 (0.7744, 0.8181)	0.8504 (0.8282, 0.8704)		0.8813 (0.8630, 0.8987)	0.7148 (0.6936, 0.7371)	–	0.7894 (0.7728, 0.8048)	0.8572 (0.8418, 0.8715)
Squamous Carcinoma		0.5692 (0.4926, 0.6434)	0.6325 (0.5577, 0.7075)	–	0.5992 (0.5356, 0.6613)	0.8912 (0.8688, 0.9138)		0.4965 (0.4470, 0.5445)	0.5595 (0.5073, 0.6116)	–	0.5261 (0.4791, 0.5662)	0.8766 (0.8606, 0.8929)
Other Malignant Tumors		0.1029 (0.0429, 0.1642)	0.2414 (0.1111, 0.3750)	–	0.1443 (0.0638, 0.2200)	0.7354 (0.6844, 0.7826)		0.1364 (0.0903, 0.1885)	0.3000 (0.2000, 0.3962)	–	0.1875 (0.1270, 0.2500)	0.7320 (0.6982, 0.7680)

CI, confidence interval; ACC, accuracy; AUC, area under the curve; pGGO, pure-ground glass opacity; mGGO, mixed-ground glass opacity; NA, not applicable.

As for the morphological characteristics, the lung cavity was identified accurately, with an AUC of 0.9176(95%CI: 0.8743-0.9553) in the validation set and 0.9074 (95%CI: 0.8834-0.9314) in the test set (**Table 2** and **Figure 3**). Spiculation, lobulation and pleural indentation reached the AUCs of 0.8466 (95%CI: 0.8236-0.8687), 0.7975 (95%CI: 0.7709-0.8204), 0.7971 (95%CI: 0.7717-0.8226) in the validation set, and of 0.8347 (95%CI: 0.8193-0.8499), 0.7818 (95%CI: 0.7642-0.7989), 0.8037 (95%CI: 0.7860-0.8203) in the test set, respectively, which could be regarded as a superior predicting performance.

### Classification Performance

**Figure 4:**
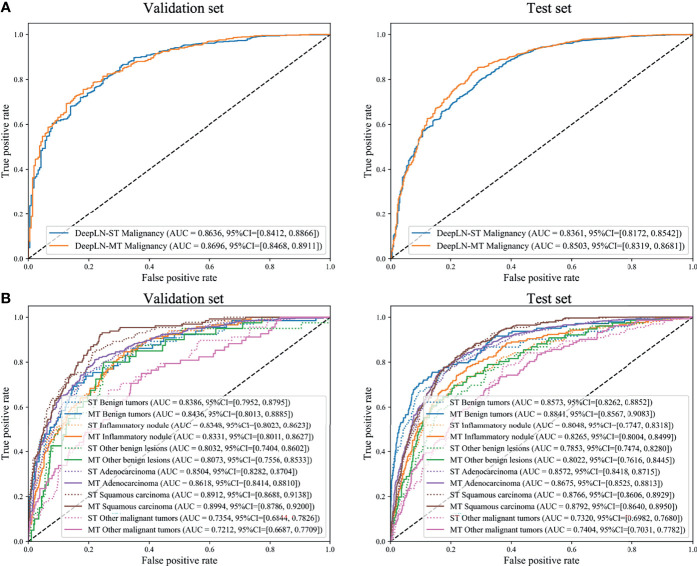
Receiver operating characteristics (ROC) curves of DeepLN to identify **(A)** malignant nodules from benign nodules and **(B)** pathological subtypes. ST, Single-task; MT, Multi-task.

**Figure 5:**
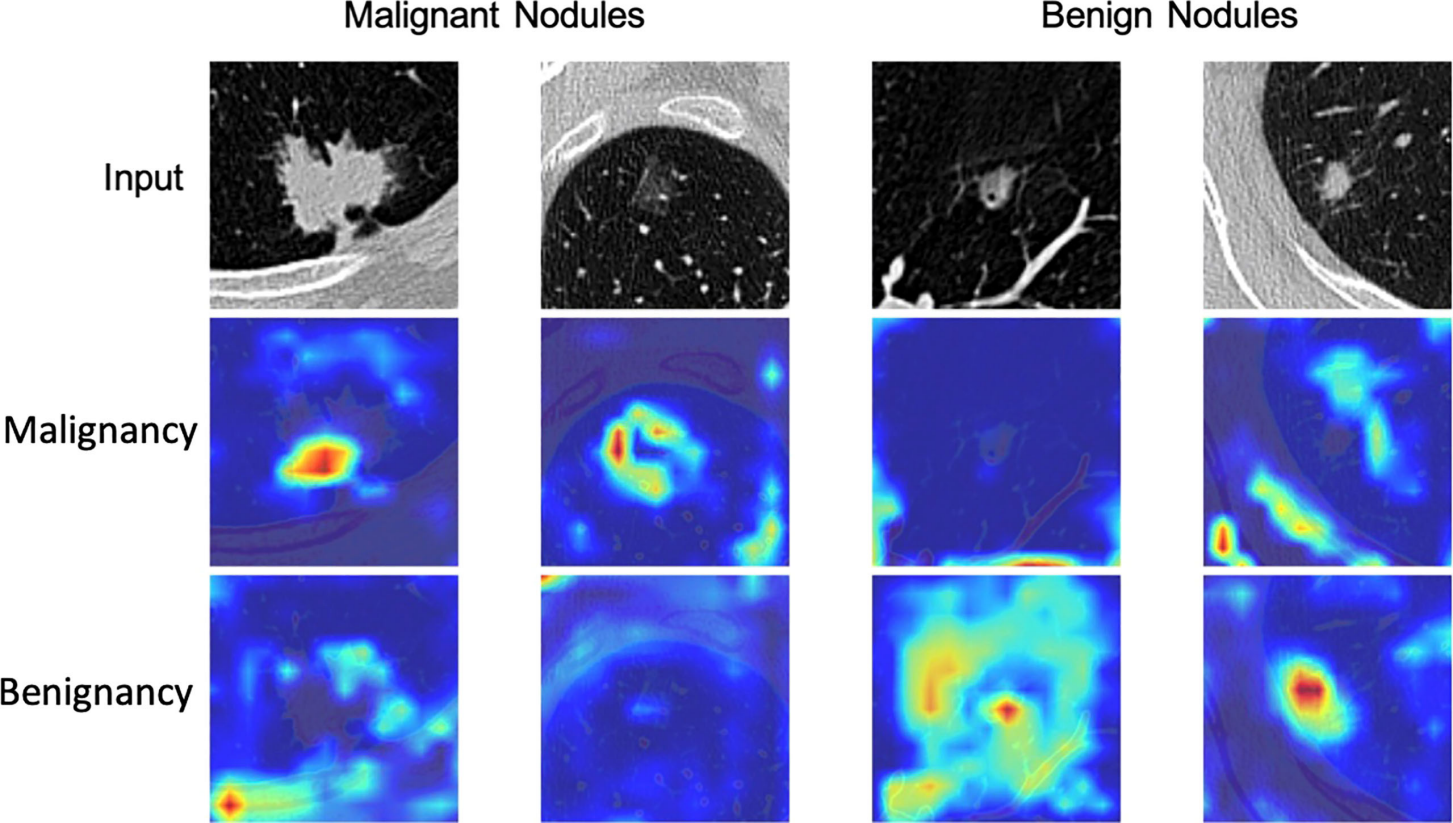
Attention map of DeepLN model in the triage of benign and malignant nodules.

The AUC of DeepLN-single task were 0.8636 (95%CI: 0.8412-0.8866), 0.8361 (95%CI: 0.8172-0.8542) for malignancy in the validation set, test set, respectively. After being fed with morphological characteristics of nodules, DeepLN achieved an excellent performance in distinguishing benign and malignant nodules (AUC = 0.8696, 95%CI: 0.8468-0.8911 in the validation set, AUC = 0.8503, 95%CI: 0.8319-0.8681 in the test set), and achieved an accuracy of 0.8331 (95%CI: 0.8185-0.8465), a precision rate of 0.8619 (95%CI: 0.8474-0.8756), and a F1 score of 0.8935 (95%CI: 0.8834-0.9034) (**Table 2** and **Figure 4**). Input crop size was defined as 20×96×96, which had superior performance compared to others sizes (**Supplementary Table 2**). In the attention maps of the deep learning model, the suspicious area with dark colors in nodules was the tumor edge and tissue between tumor and pleura (**Figure 5**).

Moreover, DeepLN could determine the specific pathological types of nodules (**Table 2**, **Figure 4** and **Supplementary Figure 1**). The performance of multi-task model was superior than single-task one, such as benign tumors (AUC=0.8436, 95%CI: 0.8013-0.8885), inflammatory ones (AUC=0.8331, 95%CI: 0.8011-0.8627), and other benign lesions (AUC=0.8073, 95%CI: 0.7556-0.8533) in the validation set. Also, different types of malignant nodules were distinguished well including LUAD (AUC=0.8618, 95%CI: 0.8414-0.8810), LUSC (AUC=0.8994, 95%CI: 0.8786-0.9200), and other cancer types (AUC=0.7212, 95%CI: 0.6687-0.7709). Then, in the test set, the performance of the model was stable with an AUC of 0.8841 (95%CI: 0.8567-0.9083) for benign tumors, 0.8265 (95%CI: 0.8004-0.8499) for inflammatory ones, and 0.8022 (95%CI: 0.7616-0.8445) for other benign lesions. For LUAD and LUSC, the AUC were 0.8675 (95%CI: 0.8525-0.8813) and 0.8792 (95%CI: 0.8640-0.8950) in the test set respectively. Even the AUC of other malignant tumors was 0.7404 (95%CI: 0.7031-0.7782).

## Discussion

LDCT-screening could investigate pulmonary nodules and detect curable early-stage lung cancer cases with a high sensitivity. Later management can highly help to improve the survival outcomes of patients with lung cancer. However, with the spread of LDCT-screening, the management of CT-detected nodules has become a growing challenging clinical problem. LDCT could screen up to 30% of participants with lung nodules, in which only 1-2% of individuals were diagnosed with lung cancer. Benign pulmonary nodules accounted for the majority of the detections (24, 25), which may result in additional investigations, unnecessary resections, and over-whelmed anxiety. Therefore, precise diagnosis and accurate pathologic identification of pulmonary nodules were of high significance, in which the adoption of automatic tools might work out to alleviate the medical burden of this workflow. For example, the number of potential benign resection rates (benign nodules identified as high risk) varying between 20% (Vanderbilt) and 30% (Oxford) datasets would potentially be suboptimal kept to a minimum (10%-20%) with the application of the LCP-CNN model (26, 27). These desirable decreases in unnecessary resections cast light on the remarkable potential of technical methods in the classification of CT-detected lung nodules.

To the best of our knowledge, our study was the first to propose a deep learning algorithm, called DeepLN, to accurately detect radiological characteristics and predict the specific pathological subtypes of pulmonary nodules with an encouraging performance. Previous attempts have merely sought to distinguish the malignant nodules from the benign ones. For instance, the lung cancer prediction convolutional neural network (LCP-CNN) could distinguish benign (low-risk) nodules from the malignant (high-risk) ones based on screening and incidentally-detected indeterminate images with encouraging performance (26, 28), in which the heterogeneity within either the malignant or benign group was ignored. In contrast, our DeepLN model accurately classified the benign nodules into different subtypes such as neoplasm, tuberculoma, inflammation, and also divided the malignant nodules into various histologic subtypes such as LUAD, LUSC and other cancers. This advancement might provided crucial guidance in everyday clinical practice. The treatment strategies varied dramatically among the different types of nodules. Some malignant nodules must require immediate surgery while others would better choose chemotherapy at the advanced stage. For example, in cases where the tumors were all with molecular alterations, the targeted therapy related to the specific genetic activations might be an ideal choice for LUAD patients, while LUSC patients with positive PD-L1 expression will choose to use immunotherapy to obtain the most favorable prognostic outcome (29). Even different benign nodules may require different solutions. Some might also entail immediate surgery to avoid harmful effects, like causing cough, and others might require regular medical treatment, such as inflammatory nodules, while others simply require interval clinic follow-ups. The detailed subtypes could play a pivotal role in the treatment decision-making, which we made great efforts to provide a novel deep learning approach to help identify.

Compared with the traditional models of lung nodule malignancy probability, such as Brock and Mayo models, deep learning was also called the “black box approach” which discovers the underlying hierarchical features invisible to naked human eyes. We used the CAM method to achieve better spatial interpretability of DeepLN and obtain the attention maps focusing on the microenvironment of nodules and pleura, which suggested that the morphology of nodules like pleural indentation was positively correlated with the risk of malignancy. This finding was consistent with those of previous studies (8, 30). Therefore, we further input imaging features with potential diagnostic values to contrast DeepLN with better classification performance. Meanwhile, this model could also detect the characteristics and density of nodules. Although the detection performance of the model was not satisfactory enough as for the mGGOs probably due to the subjectivity of annotation and several potential errors, the overall performance of our ompetetive with the aforementioned careful design.

In addition, the whole development, training, adjusting, test, and validating processes of our model were based on a so-far largest clinical dataset of non-invasive CT images of lung nodules adopted to address a relative problem in the lung cancer field. We had a massive coverage of patients and nodules. A total of 8950 lung nodules from 5823 patients were identified and included in our study, of which there were 2211 benign nodules and 6739 malignancy nodules. Our model achieved a promising performance on the basis of such a reliable dataset in the prediction of the histological subtypes when the pathology results were referred to the gold standard. Recent studies have reported related radiomic and deep machine learning methods that exhibit the human-level performance in predicting the malignant risk of pulmonary nodules, but most of these studies trained on the model *via* a small dataset or available public dataset without pathologic results such as NLST based on Lung-RADS risk bucket (14, 31, 32). Compared with these previous studies, our results provided a more comprehensive, reliable, holistic and heterogeneous automatic tool in the subtype classification of lung nodule cases developed based on real-world clinical data and might be of better clinical value, especially for the Asian population.

There were several limitations in the current study. First, the biases were inevitable due to the retrospective nature of this study from a single center, and thus further multicentric and prospective investigations would be needed. Second, most of the pathology-diagnosed nodules were more than 8 mm in diameter in the current study, so there might be smaller sized nodules that were neglected. But our algorithm could exhibit a great performance in the detection in the small nodules less than 8 mm (17, 18), so the exploration might be properly extended. Third, our study showed that 24.70% of the nodules were benign, which was far less than expected. In the whole patient population, benign cases comprised the majority of all the pulmonary nodules detected, which made training the reliable classification model vital to avoid unnecessary biopsy or surgery. The inclusion of patients limited the general applicability of our model. Its robustness and generality should be further validated in more representative cohorts.

This study provided a promising diagnostic tool named DeepLN that could identify the radiological morphological manifestations such as spiculation, lobulation or pleural indentation, as well as differentiate the malignant tumors (such as LUAD and LUSC) from the benign lesions (such as neoplasm and inflammation). All the classification processes conducted by the proposed DeepLN algorithm were based on chest CT scans, offering a noninvasive and reproducible solution to define the histological subtypes of lung nodules. Furthermore, the morphological features that contribute most to our model can be investigated. Our method could also be extended in larger, multicentric, prospective randomized studies to verify and enhance its superiority in stratifying the subtypes and predicting the actual clinical outcomes of patients with CT-detected nodules.

## Data Availability Statement

The raw data supporting the conclusions of this article will be made available by the authors, without undue reservation.

## Author Contributions

WL and ZY conceived and designed the study. CW, JS, and YH contributed to data analysis and preparation of the report. XX, LY, JG, CB, and LZ collected samples and assembled the imaging and clinical data. XX and LY conducted statistical analysis. CW, JS, and XX wrote drafted the manuscript. All authors read and approved the final manuscript.

## Funding

This study was supported by grants 82100119, 91859203, 92159302 from National Natural Science Foundation of China, grant 2018AAA0100201 from National Major Science and Technology Projects of China, grant 2020YFG0473 and 2022ZDZX0018 from the Science and Technology Project of Sichuan, and 2021M692309 from Chinese Postdoctoral Science Foundation.

## Conflict of Interest

The authors declare that the research was conducted in the absence of any commercial or financial relationships that could be construed as a potential conflict of interest.

## Publisher’s Note

All claims expressed in this article are solely those of the authors and do not necessarily represent those of their affiliated organizations, or those of the publisher, the editors and the reviewers. Any product that may be evaluated in this article, or claim that may be made by its manufacturer, is not guaranteed or endorsed by the publisher.

## Supplementary Material

Supplementary Figure 1 Confusion matrix of DeepLN to identify **(A)** density of nodules, **(B)** malignancy nodules from benign nodules, **(C)** six subtypes(1, benign tumors; 2, inflammatory nodules; 3, other benign lesions; 4, adenocarcinoma; 5, squamous carcinoma; 6, other malignant tumors).

Supplementary Table 1 CLAIM: Checklist for Artificial Intelligence in Medical Imaging.

Supplementary Table 2 Performance of DeepLN to classify benign and malignant lung nodules with different input crop sizes.
