# Supplementary figures and images for: DeepLN: A Multi-Task AI Tool to Predict the Imaging Characteristics, Malignancy and Pathological Subtypes in CT-Detected Pulmonary Nodules
Source: Front Oncol. 2022 May 11;12:683792. doi: 10.3389/fonc.2022.683792 (PMC9130467; doi:10.3389/fonc.2022.683792)

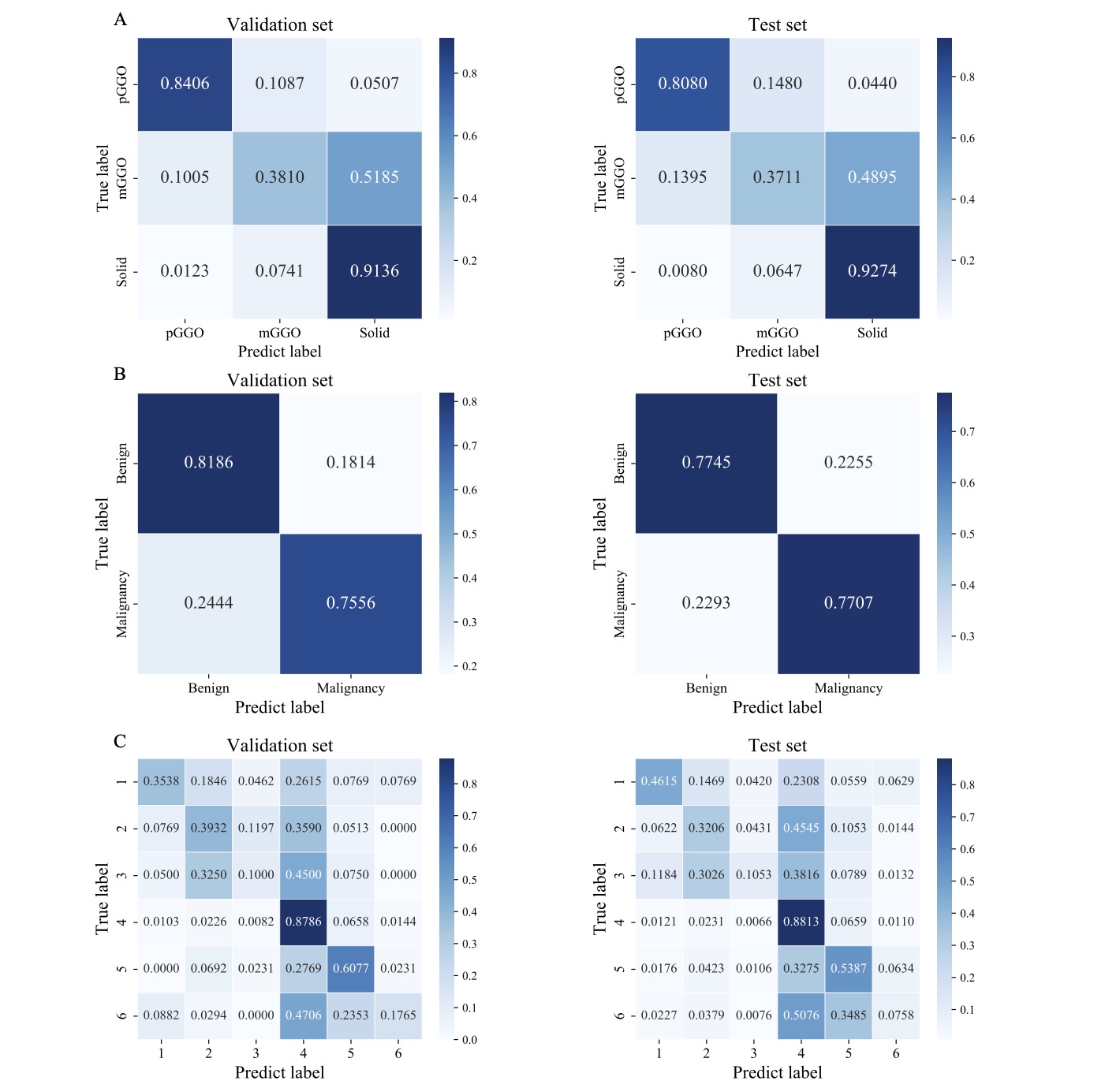

Supplement: Supplementary Figure 1 — Confusion matrix of DeepLN to identify (A) density of nodules, (B) malignancy nodules from benign nodules, (C) six subtypes(1, benign tumors; 2, inflammatory nodules; 3, other benign lesions; 4, adenocarcinoma; 5, squamous carcinoma; 6, other malignant tumors). [file Image_1.jpeg]
