# Supplementary material for: DeepLN: A Multi-Task AI Tool to Predict the Imaging Characteristics, Malignancy and Pathological Subtypes in CT-Detected Pulmonary Nodules
Source: Front Oncol. 2022 May 11;12:683792. doi: 10.3389/fonc.2022.683792 (PMC9130467; doi:10.3389/fonc.2022.683792)
Supplement: Supplementary Table 1 — CLAIM: Checklist for Artificial Intelligence in Medical Imaging. [file Table_1.docx]

**Table S1. CLAIM: Checklist for Artificial Intelligence in Medical Imaging**

| **Section / Topic** | **No.** | **Item** |  |
| --- | --- | --- | --- |
| TITLE / ABSTRACT |  |  |  |
|  | **1** | Identification as a study of AI methodology, specifying the category of technology used (e.g., deep learning) | Confirmed |
|  | **2** | Structured summary of study design, methods, results, and conclusions | Confirmed |
| INTRODUCTION |  |  |  |
|  | **3** | Scientific and clinical background, including the intended use and clinical role of the AI approach | Confirmed |
|  | **4** | Study objectives and hypotheses | Confirmed |
| METHODS |  |  |  |
| *Study Design* | **5** | Prospective or retrospective study | Retrospective cohort |
|  | **6** | Study goal, such as model creation, exploratory study, feasibility study, non-inferiority trial | Confirmed |
| *Data* | **7** | Data sources | Confirmed |
|  | **8** | Eligibility criteria: how, where, and when potentially eligible participants or studies were identified (e.g., symptoms, results from previous tests, inclusion in registry, patient-care setting, location, dates) | Confirmed |
|  | **9** | Data pre-processing steps | Confirmed |
|  | **10** | Selection of data subsets, if applicable | NA |
|  | **11** | Definitions of data elements, with references to Common Data Elements | Confirmed |
|  | **12** | De-identification methods | Confirmed |
|  | **13** | How missing data were handled | NA |
| *Ground Truth* | **14** | Definition of ground truth reference standard, in sufficient detail to allow replication | Confirmed |
|  | **15** | Rationale for choosing the reference standard (if alternatives exist) | Confirmed |
|  | **16** | Source of ground-truth annotations; qualifications and preparation of annotators | Confirmed |
|  | **17** | Annotation tools | Confirmed |
|  | **18** | Measurement of inter- and intrarater variability; methods to mitigate variability and/or resolve discrepancies | Confirmed |
| *Data Partitions* | **19** | Intended sample size and how it was determined | NA |
|  | **20** | How data were assigned to partitions; specify proportions | Confirmed |
|  | **21** | Level at which partitions are disjoint (e.g., image, study, patient, institution) | Confirmed |
| *Model* | **22** | Detailed description of model, including inputs, outputs, all intermediate layers and connections | Confirmed |
|  | **23** | Software libraries, frameworks, and packages | Confirmed |
|  | **24** | Initialization of model parameters (e.g., randomization, transfer learning) | Confirmed |
| *Training* | **25** | Details of training approach, including data augmentation, hyperparameters, number of models trained | Confirmed |
|  | **26** | Method of selecting the final model | Confirmed |
|  | **27** | Ensembling techniques, if applicable | NA |
| *Evaluation* | **28** | Metrics of model performance | Confirmed |
|  | **29** | Statistical measures of significance and uncertainty (e.g., confidence intervals) | Confirmed |
|  | **30** | Robustness or sensitivity analysis | Confirmed |
|  | **31** | Methods for explainability or interpretability (e.g., saliency maps), and how they were validated | Confirmed |
|  | **32** | Validation or testing on external data | NA |
| RESULTS |  |  |  |
| *Data* | **33** | Flow of participants or cases, using a diagram to indicate inclusion and exclusion | NA |
|  | **34** | Demographic and clinical characteristics of cases in each partition | Confirmed |
| *Model performance* | **35** | Performance metrics for optimal model(s) on all data partitions | Confirmed |
|  | **36** | Estimates of diagnostic accuracy and their precision (such as 95% confidence intervals) | Confirmed |
|  | **37** | Failure analysis of incorrectly classified cases | Confirmed |
| DISCUSSION |  |  |  |
|  | **38** | Study limitations, including potential bias, statistical uncertainty, and generalizability | Confirmed |
|  | **39** | Implications for practice, including the intended use and/or clinical role | Confirmed |
| OTHER INFORMATION |  |  |  |
|  | **40** | Registration number and name of registry | Confirmed |
|  | **41** | Where the full study protocol can be accessed | Confirmed |
|  | **42** | Sources of funding and other support; role of funders | Confirmed |

**Reference**

Mongan J, Moy L, Kahn CE, Jr. Checklist for Artificial Intelligence in Medical Imaging (CLAIM): A Guide for Authors and Reviewers. *Radiol Artif Intell* (2020) 2(2):e200029. Epub 2020/03/25. doi: 10.1148/ryai.2020200029.

**Table S2. Performance of DeepLN to classify benign and malignant lung nodules with different input crop sizes.**

|  | Validation Set | | | | | | Test Set | | | | | |
| --- | --- | --- | --- | --- | --- | --- | --- | --- | --- | --- | --- | --- |
| Input crop size | **ACC** | **Sensitivity** | **Precision** | **Specificity** | **F1 score** | **AUC** | **ACC** | **Sensitivity** | **Precision** | **Specificity** | **F1 score** | **AUC** |
| 20×48×48 | 0.8424 | 0.9385 | 0.8646 | 0.5442 | 0.9001 | 0.8533 | 0.8197 | 0.9223 | 0.8512 | 0.5034 | 0.8853 | 0.838 |
| 20×64×64 | 0.8299 | 0.9265 | 0.8595 | 0.5302 | 0.8918 | 0.8595 | 0.8252 | 0.9216 | 0.8575 | 0.5285 | 0.8884 | 0.8409 |
| 20×96×96 | 0.8356 | 0.925 | 0.8666 | 0.5581 | 0.8949 | 0.8696 | 0.8331 | 0.9275 | 0.8619 | 0.5421 | 0.8935 | 0.8503 |
| 20×128×128 | 0.8209 | 0.9235 | 0.852 | 0.5023 | 0.8863 | 0.8432 | 0.8096 | 0.9149 | 0.8455 | 0.4852 | 0.8789 | 0.8035 |
